# Citrullinated Antigens with Multiple Citrulline Similar Motif in the Diagnosis of Rheumatoid Arthritis: A Preliminary Single-Center Study

**DOI:** 10.1155/2021/1891519

**Published:** 2021-08-10

**Authors:** Zhu Chen, Weiting Fang, Yi Qin, Yin-Zhao Jin, Xiang Yang, Jianrong Lou

**Affiliations:** ^1^Department of Rheumatology and Immunology, Anhui Medical University Affiliated Provincial Hospital, Lujiang Str 17, Hefei 230001, China; ^2^Department of Rheumatology and Immunology, The First Affiliated Hospital of USTC, Division of Life Sciences and Medicine, University of Science and Technology of China, Hefei 230001, China; ^3^Leide Biosciences Co., Ltd., Guangzhou, China

## Abstract

The presence of anti-citrullinated protein antibodies (ACPAs) in the serum is one of the immunological features of rheumatoid arthritis (RA). Anti-cyclic citrullinated peptide (CCP) assay has been widely used in clinic for the diagnosis of RA. However, up to 40% of RA patients are anti-CCP negative and the diagnostic sensitivity in this population needs to be improved for better clinical management. In this study, peptides with Multiple Citrulline Similar Motif (MCSM) were synthesized and a new ELISA system, which we called RA_CP, was developed to detect citrullinated antigens with MCSM present in the serum. 106 RA,48 other arthritis patients and 41 sex- and age-matched healthy controls (HCs) were included in this study. Patients with RA have a significantly higher amount of citrullinated antigens with MCSM than other arthritis patients and HCs. RA patients with positive anti-CCP are also MCSM positive, whereas 75% anti-CCP negative patients are positive for MCSM. The diagnostic sensitivity for anti-CCP and MCSM was 81.1% and 95.3%, while the specificity was 100% and 94.4%, respectively. ROC curve analyses showed that the area under the curve (AUC) values were 0.906 (95% CI: 0.860-0.951) for anti-CCP and 0.948 (95% CI: 0.912-0.985) for MCSM while the combination of MCSM and anti-CCP test has the highest AUC (0.971, 95% CI: 0.946–0.996). Our results suggest that detection of citrullinated antigens with MCSM has improved sensitivity compared with anti-CCP assay and could serve as a biomarker in diagnosis of RA patients.

## 1. Introduction

Rheumatoid arthritis (RA) is a chronic autoimmune-mediated inflammatory disease which mainly affects small to medium joints and remains one of the leading courses of disability in western countries [1]. Early diagnosis and sufficient treatment of the disease are crucial to prevent joint damage and irreversible disability. Although progression has been made regarding diagnosis of RA with the 2010 classification criteria developed by ACR/EULAR, there are still clinical unmet needs to find out more sensitive serological biomarkers for early diagnosis and intervention of RA [2, 3].

One of the immunological features of RA is the presence of autoantibodies to immunoglobulin G (rheumatoid factor (RF)) and citrullinated proteins (anti-citrullinated protein antibodies (ACPAs)). It is now well established that ACPAs are not only a specific diagnostic marker but also a predictor for more aggressive disease course and poor prognosis [4–7]. Accordingly, anti-cyclic citrullinated peptide (CCP) detected by ELISA has been introduced and widely used in clinic, serving as an important biomarker for diagnosis of RA [8]. However, up to 40% of RA patients are anti-CCP negative and the diagnostic sensitivity in this population needs to be improved for better clinical management [9, 10]. Considering antigens normally emerge earlier than antibodies, we hypothesize it would be promising to detect citrullinated antigens for early diagnosis of RA patients. In this study, peptides with Multiple Citrulline Similar Motif (MCSM) were synthesized and used to produce anti-MCSM antibody. A new ELISA system, which we called RA_CP, was then developed to detect citrullinated antigens with MCSM present in serum of RA patients. We further examined the sensitivity and specificity of citrullinated antigens with MCSM and compared with conventional anti-CCP test to evaluate its potential value for application in clinic.

## 2. Materials and Methods

### 2.1. Patients

RA patients aged >18 years who met the 2010 ACR/EULAR classification criteria [2] were enrolled from Anhui Medical University Affiliated Provincial Hospital from November 2019 to December 2020. Patients with concomitant autoimmune diseases such as systemic lupus erythematosus and Sjogren's syndrome were excluded. Demographic features, clinical manifestations, and laboratory parameters including erythrocyte sedimentation rate (ESR), C-reactive protein (CRP), RF, and anti-CCP were collected and recorded. Disease Activity Score-28 (DAS28) was calculated to evaluate disease activity. In addition, patients with other types of arthritis who visited the clinic during the same period and sex- and age-matched healthy individuals were selected as control. This study was approved by the ethic committee of Anhui Medical University Affiliated Provincial Hospital.

### 2.2. Synthesis of Peptides with MCSM

**Figure 1:**
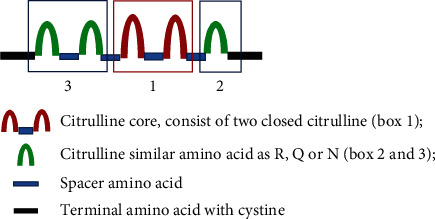
Multiple Citrulline Similar Motif (MCSM) structure. R: arginine; Q: glutamine; N: asparagine.

Peptides were designed based on the MCSM motif, which is composed of the citrulline core and multicitrulline similar amino acid structures around the citrulline core. The MCSM motif structure is shown in Figure 1. The sequence of the peptide used for immunization is GCGGRSQFNW(Cit)S(Cit)SRPRGCGG. Peptides were synthesized, cycled, and purified by GL Biochem (Shanghai) Ltd.

### 2.3. Development and Identification of Anti-MCSM Antibody

Two NZW SPF rabbits (New Zealand White rabbits) were used for immunization. Peptide was emulsified in the Complete Freund's Adjuvant (CFA) and injected subcutaneously. A total of 4 times of immunization were executed and 72 days was used for antibody production. Anti-MCSM antibody was affinity purified by protein A resin, and reactivity of anti-MCSM with MCSM or control peptide was examined by ELISA. Briefly, 96-well plates (Nunc) were coated with MCSM peptide and control peptide, respectively, overnight at 2~8°C. After washing, gradient-diluted anti-MCSM antibody was added and incubated for 1 hour, followed by washing and incubation with goat anti-rabbit IgG antibody/HRP for 0.5 h. The OD_450_ value was measured with a microplate reader.

### 2.4. Western Blotting

The MCSM peptide and control peptide were subjected to SDS-PAGE and transferred to a polyvinylidene fluoride membrane (Millipore). After blocking with 5% skim milk/PBST for 30 minutes, the membrane was incubated with 10 *μ*g/ml biotin-conjugated anti-MCSM antibody for 1 hour then successively with 1 : 5000 streptavidin/HRP for 1 hour after washing with PBST.

### 2.5. RA_CP ELISA

RA_CP ELISA was developed based on anti-MCSM antibody and provided by Leide Bioscience (Cat# 2001-96). Briefly, 96-well plates (Nunc) were coated with anti-MCSM antibody. Serum samples diluted at 1 : 10 with or without protein A/G resin treatment were added to the well and incubated at room temperature for 1 hour. After being washed three times, primary antibody and HRP-conjugated secondary antibody were added and incubated for 1 hour at room temperature. Absorbance was measured at 450 nm. A cut-off value discriminating between MCSM-positive and MCSM-negative patients was based on absorbance ratio (sample OD value/critical control OD value). Absorbance ratio < 0.95 was thought as MCSM negative, whereas absorbance ratio > 1.05 was defined as MCSM positive.

### 2.6. Statistical Analyses

All data were presented as mean ± standard deviation (SD) or median with quartile (P25, P75). For quantitative parameters, differences among groups were assessed by one-way ANOVA followed by Bonferroni's multiple-comparisons test. Categorical variables were reported as counts (percentage) and compared using Fisher's exact test. Receiver operating characteristic (ROC) curve was performed to compare the diagnostic performance of the tests and analysis of sensitivity and specificity. Kendall's tau-b correlation analysis was used to explore association of MCSM with clinical and laboratory parameters. All analyses were performed by SPSS 26. Two-tailed *P* values of 0.05 or less were considered as statistically significant.

## 3. Results

### 3.1. Reactivity of Anti-MCSM Antibody with Citrullinated Peptide

**Figure 2:**
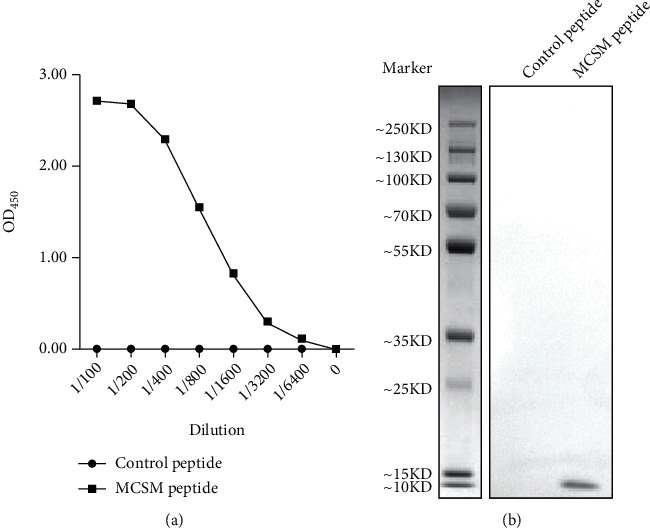
Reactivity of anti-MCSM antibody with citrullinated peptide. (a) Binding of anti-MCSM antibody with MCSM peptide in ELISA. (b) Reactivity of anti-MCSM antibody with citrullinated peptide was identified by western blotting.

To confirm that anti-MCSM antibody really reacts with citrullinated peptide, we coated microplates with MCSM or control peptide and incubated with gradient diluted anti-MCSM antibody. As shown in Figure 2(a), anti-MCSM at 1 : 100 to 1 : 6400 dilution reacts with MCSM but not control peptide. This specific binding was further confirmed by western blotting (Figure 2(b)).

### 3.2. The Clinical and Demographical Characteristics of RA Patients

A total of 106 patients with RA, 48 other arthritis patients (26 spondyloarthritis, 21 osteoarthritis, and 1 juvenile idiopathic arthritis), and 41 sex- and age-matched healthy subjects were included in this study. Among the 106 patients with RA, the mean age was 57 years and 78% were female. 76 (77%) patients were positive with RF and 86 (81%) were anti-CCP positive. The clinical and demographical features of RA patients are listed in Table 1.

### 3.3. Prevalence of Citrullinated Antigens with MCSM in Different Disease Cohorts

**Figure 3:**
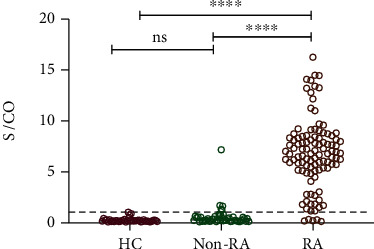
Quantification of citrullinated antigens with MCSM in serum by ELISA. ^∗∗∗∗^*P* < 0.0001 (one-way ANOVA followed by Bonferroni's multiple-comparisons test).

Using RA_CP ELISA to detect citrullinated antigens against anti-MCSM antibody, we investigated sera from 106 RA patients, 48 other arthritis patients, and 41 sex- and age-matched healthy controls (HCs). 101 out of the 106 RA patients (95.3%) and 5 out of the 48 non-RA patients (10.4%, including 3 ankylosing spondylitis, 1 osteoarthritis, and 1 undifferentiated spondyloarthritis) were positive for citrullinated antigens with MCSM. All of the HCs were negative in the test. Patients with RA showed significantly higher amount of citrullinated antigens with MCSM than other arthritis patients and HCs (Figure 3). Notably, when serum with positive MCSM and anti-CCP was pretreated by protein A/G resins, significant MCSM signals remained in RA_CP ELISA, whereas no signal was found in the elution of anti-CCP-positive serum (Supplemental Figure 1), suggesting the specificity of RA_CP ELISA for detecting citrullinated antigens.

For further analyses, RA patients were divided into RF positive, RF negative, anti-CCP positive, and anti-CCP negative. As expected, patients with positive RF and anti-CCP had significantly higher percentage of serum MCSM than patients with negative RF and anti-CCP, suggesting that citrullinated antigens are associated with both RF and anti-CCP antibody (Table 2).

### 3.4. Sensitivity and Specificity of Citrullinated Antigens with MCSM in Diagnosis of RA

**Figure 4:**
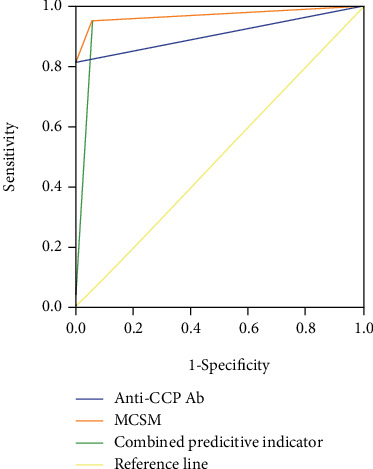
Comparative receiver operating characteristic (ROC) curve analysis for anti-CCP and MCSM and combination test.

In order to compare the diagnostic performance of the tests, ROC curve analysis was performed for anti-CCP and citrullinated antigens with MCSM. As shown in Figure 4, the diagnostic sensitivity for anti-CCP and MCSM was 81.1% and 95.3%, respectively, while the specificity was 100% and 94.4%, respectively. The area under the curve (AUC) values were 0.906 (95% CI: 0.860-0.951) for anti-CCP and 0.948 (95% CI: 0.912-0.985) for MCSM. This result suggests that detection of citrullinated antigens with MCSM has an improved sensitivity but lower specificity for diagnosis of RA, compared with anti-CCP antibody.

Next, we explored whether a combination of the two tests has a better performance for diagnosing RA. Binary logistic regression model was performed to calculate the joint factor model (Figure 4). We observed that the combination of MCSM and anti-CCP test led to the highest AUC (0.971), compared with using the values of MCSM or anti-CCP alone (Table 3), suggesting that combination test has a better performance in early diagnosis of RA. The sensitivity and specificity were 95.28% and 94.38% for the combined predictive indicator.

### 3.5. Clinical Implication of Citrullinated Antigens with MCSM in RA

To investigate whether elevated levels of citrullinated antigens with MCSM have clinical implications in RA, we explored correlation analyses of serum MCSM with clinical and laboratory parameters. Interestingly, we found a positive correlation of MCSM with RF titer, while no associations of ESR, CRP, and DAS28 were observed (Table 4).

Since 15 out of 20 RA patients with negative anti-CCP were positive for MCSM, we further compared the clinical characteristics of MCSM-positive and MCSM-negative patients with negative anti-CCP. As shown in Table 5, we did not observe differences in disease duration, age, ESR, CRP, and DAS28 between these two populations.

## 4. Discussion

The conversion of an arginine residue in a protein to a citrulline residue by peptidylarginine deiminases (PADs) is a key process in the immunopathogenesis of RA [11, 12]. ACPA, which recognizes citrullinated autoantigens such as filaggrin, collagen type II, vimentin, and fibrinogen, has been thought as one of the most important serological as well as prognostic markers of RA [13]. As a result, the anti-CCP ELISA test was developed since 1998 for the more convenient and accurate identification of ACPA-positive RA patients [8]. More recently, the second and third generations of anti-CCP tests were developed to increase the diagnostic performance for RA [14]. Although the sensitivity has been improved with the modified anti-CCP tests, more than 40% RF-negative RA patients were also negative in anti-CCP3 test [15].

The success of the anti-CCP assay in the diagnosis of RA has led to the effort to identify additional citrullinated autoantigens [16]. Based on previous knowledge on CCP-related peptide sequence, we designed and synthesized a multicitrulline similar amino acid motif (MCSM), which comprises a citrulline core and multicitrulline similar amino acid structures around the citrulline core. For the first time, we utilized an anti-MCSM antibody to detect MCSM in the serum of RA patients by ELISA. Our preliminary results showed that, compared with the anti-CCP assay, the MCSM assay has significantly improved sensitivity, while it maintained high specificity for the diagnosis of RA. When MCSM and anti-CCP assay were combined, it reaches the highest AUC in ROC curve analyses, although sensitivity and specificity did not increase accordingly.

Among all the RA patients tested, patients with positive anti-CCP are also MCSM positive, whereas 75% anti-CCP negative patients are positive for MCSM, which suggests that citrullinated antigens are widespread in RA and detection of MCSM is very promising in early diagnosis for these anti-CCP-negative RA patients. However, among the anti-CCP-negative patients, we did not find a significant difference between MCSM-positive and MCSM-negative patients, which might be explained by the limited number of samples. It would be interesting to follow up these patients and investigate how the citrullinated antigens with MCSM are distributed in inflammatory sites such as synovial fluid. Considering anti-CCP antibody was associated with more aggressive disease course and bone loss in RA, we are planning to assess whether MCSM is also a marker for poor prognosis in the next prospective studies.

In our study, we further explored the association of MCSM with clinical and laboratory features of RA patients. We only find a positive correlation of RF with MCSM. This is interesting since a recent research found that IgG ACPA-IgM RF immune complexes are present in the serum of RA patients [17], although another research indicated ACPA^+^ and RF^+^ B cells express distinct transcriptional programs [18].

## 5. Conclusions

In summary, our preliminary study suggested that detection of citrullinated antigens with MCSM is promising in diagnosis of RA patients, especially those with negative anti-CCP antibody. Further research on larger cohort and prospective study in pre-RA patients are needed to validate the efficiency in clinical settings.

## Figures and Tables

**Table 1:** The clinical and demographical characteristics of RA patients.

Parameters	Mean (% or quartile)
Age (years)	57 (47.0-67.3)
Female	83 (78%)
Disease duration (years)	8 (1.5-15.0)
ESR (mm/h)	55.5 (26.8-77)
CRP (mg/l)	10.6 (3.3-52.7)
RF (IU/ml)	86.5 (21.7-283.0)
RF positive	76 (77%)
Anti-CCP positive	86 (81%)
DAS28-ESR	4.9 (3.6-6.0)
SJC	1 (0-2.3)
TJC	2 (1-8)

ESR: erythrocyte sediment rate; CRP: C-reactive protein; SJC: swollen joint count; TJC: tender joint count; DAS28: Disease Activity Score-28; RF: rheumatoid factor; CCP: cyclic citrullinated peptide.

**Table 2:** MCSM in RA patients with or without RF and anti-CCP.

Group	MCSM negative (*n*)	MCSM positive (*n*)	*P* value
RF negative	5	19	0.001
RF positive	0	77	
Anti-CCP negative	5	15	<0.001
Anti-CCP positive	0	86	

**Table 3:** Evaluation of the diagnostic value of predictive indicators for RA.

	AUC	95% CI	*P* value
Anti-CCP	0.906	0.860–0.951	<0.001
MCSM	0.948	0.912–0.985	<0.001
Combined predictive indicator	0.971	0.946–0.996	<0.001

**Table 4:** Correlation analysis of clinical data and citrullinated antigen in RA patients.

Parameters	*r*	*P* value
Age	-0.011	0.869
Disease duration	0.056	0.408
ESR	0.073	0.260
CRP	0.088	0.182
RF	0.331	<0.001
DAS28	0.111	0.087

**Table 5:** Comparison of MCSM^+^ and MCSM^−^ patients with negative anti-CCP.

Group	*n*	Course	Age	ESR	CRP	DAS28
MCSM^+^anti-CCP^−^	15	6 (2, 11)	65 (55, 72)	32 (20, 64.5)	6.28 (3.11, 29.5)	4.76 ± 1.67
MCSM^−^anti-CCP^−^	5	10 (6.5, 22.5)	63 (51, 76.5)	59 (32.5, 92.5)	21 (7.29, 68.9)	4.55 ± 1.78
*Z*		-1.359	-0.44	-1.266	-1.181	-0.218
*P*		0.174	0.965	0.205	0.238	0.827

## Data Availability

Data will be available from authors on request.

## Supplementary Materials

Supplementary Materials Supplemental Figure 1: pretreatment of serum from 6 RA patients and 2 HCs by protein A/G resin.
