# Supplementary material for: Citrullinated Antigens with Multiple Citrulline Similar Motif in the Diagnosis of Rheumatoid Arthritis: A Preliminary Single-Center Study
Source: J Immunol Res. 2021 Aug 10;2021:1891519. doi: 10.1155/2021/1891519 (PMC8376434; doi:10.1155/2021/1891519)
Supplement: Supplementary Materials — Supplemental Figure 1: pretreatment of serum from 6 RA patients and 2 HCs by protein A/G resin. [file 1891519.f1.docx]

**Supplemental Figure 1.**


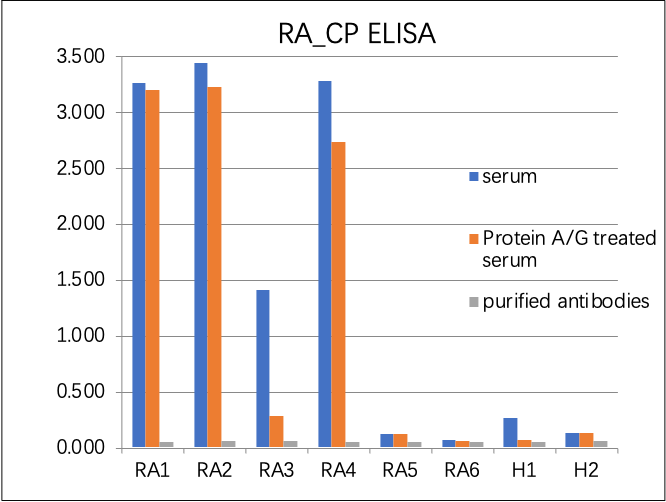


OD_450_

Supplemental Figure 1. Pre-treatment of serum from 6 RA patients and 2 HCs by protein A/G resin. RA_CP ELISA was set up to measure citrullinated antigens with MCSM in the serum with or without protein A/G resin treatment. RA1 and RA2: patients with MCSM and anti-CCP double positive. RA3 and RA4: anti-CCP negative and MCSM positive. RA5 and RA6: anti-CCP positive and MCSM negative. H1 and H2: anti-CCP and MCSM double negative healthy controls.
